# Cytokine-based models for efficient differentiation between infection and cytokine release syndrome in patients with hematological malignancies

**DOI:** 10.1186/s40164-024-00495-6

**Published:** 2024-03-05

**Authors:** Linqin Wang, Yuqi Lv, Linghui Zhou, Shenghao Wu, Yuanyuan Zhu, Shan Fu, Shuyi Ding, Ruimin Hong, Mingming Zhang, Hanjing Yu, Alex H. Chang, Guoqing Wei, Yongxian Hu, He Huang

**Affiliations:** 1 Bone Marrow Transplantation Center, The First Affiliated Hospital, Zhejiang University School of Medicine, Hangzhou, China; 2 Liangzhu Laboratory, Zhejiang University, Hangzhou, China; 3 Institute of Hematology, Zhejiang University, Hangzhou, China; 4 Zhejiang Province Engineering Research Center for Stem Cell and Immunity Therapy, Hangzhou, China; 5 Department of Hematology, The Dingli Clinical College of Wenzhou Medical University (The Second Affiliated Hospital of Shanghai University, Wenzhou Central Hospital), Wenzhou, China; 6 Hangzhou Medical College, Hangzhou, China; 7 Shanghai YaKe Biotechnology Ltd., Shanghai, China; 8 Engineering Research Center of Gene Technology, Ministry of Education, Institute of Genetics, School of Life Sciences, Fudan University, Shanghai, China

**Keywords:** Cytokine release syndrome, Infection, Fever, Differentiation models, Chimeric antigen receptor, Hematological malignancies

## Abstract

Although the efficacy of chimeric antigen receptor (CAR)-T cell therapy has been widely demonstrated, its clinical application is hampered by the complexity and fatality of its side effects. Cytokine release syndrome (CRS) is the most common toxicity following CAR-T cell infusion, and its symptoms substantially overlap with those of infection. Whereas, current diagnostic techniques for infections are time-consuming and not highly sensitive. Thus, we are aiming to develop feasible and efficient models to optimize the differential diagnosis in clinical practice. This study included 191 febrile patients from our center, including 85 with CRS-related fever and 106 with infectious fever. By leveraging the serum cytokine profile at the peak of fever, we generated differential models using a classification tree algorithm and a stepwise logistic regression analysis, respectively. The first model utilized three cytokines (IFN-β, CXCL1, and CXCL10) and demonstrated high sensitivity (90% training, 100% validation) and specificity (98.44% training, 90.48% validation) levels. The five-cytokine model (CXCL10, CCL19, IL-4, VEGF, and CCL20) also showed high sensitivity (91.67% training, 95.65% validation) and specificity (98.44% training, 100% validation). These feasible and accurate differentiation models may prompt early diagnosis of infections during immune therapy, allowing for early and appropriate intervention.

**Supplementary Information:**

The online version contains supplementary material available at 10.1186/s40164-024-00495-6.

To the editor,

Though chimeric antigen receptor (CAR)-T cell therapy has largely compensated for the limitations of conventional treatments for relapsed or refractory (r/r) hematological malignancies (HMs), the extensive clinical application of this therapy has been impeded by the occurrence of severe or potentially fatal toxicity [1–3]. Cytokine release syndrome (CRS), one of the most common toxicities, exhibits non-specific manifestations such as elevated cytokine levels and high fever, which can complicate clinical diagnosis and intervention [1, 4–6]. Meanwhile, patients who undergo CAR-T cell infusion (CTI) frequently encounter severe cytopenia, B-cell aplasia, and/or hypogammaglobulinemia, rendering them susceptible to infections that bear tremendous resemblances to CRS [7–10]. It’s noteworthy that corticoid-based regimens employed for CRS management differ significantly from anti-infection strategies and may potentially exacerbate infections. Without prompt intervention, infections may escalate uncontrollably. Given the limitations of conventional diagnostic techniques in terms of sensitivity and immediacy, we developed two cytokine-based models for efficient differentiation between infection and CRS in a large cohort of patients with HMs.

A total of 191 patients with HM suffering from fever were followed. Among them, eighty-five patients had a fever attributed to CRS, while 106 had a fever as a response to infections. The CRS cohort consisted of 72 patients (84.71%) with r/r multiple myeloma and 13 patients (15.29%) with r/r leukemia. Following CTI, forty-seven patients (55.29%) experienced severe CRS, while 38 (44.71%) developed non-severe CRS (Fig. 1A). Additionally, the infection cohort consisted of 26 patients who experienced fever after CTI and 80 patients without a history of CTI. All cases were confirmed by microbial etiology. Among them, multiple infections were observed in 28 patients (26.42%). Among the remaining cases, single-pathogen infections were observed in 78 patients, including gram-negative bacterial infections (36/106, 33.96%), fungal infections (24/106, 22.64%) and gram-positive bacterial infections (18/106, 16.98%) (Fig. 1B). Regarding the site of infection, seven patients suffered from infections at multiple sites, while the others developed single-site infections. In cases of single-site infections, the digestive tract was the most frequently affected site (32/106, 30.19%), followed by the bloodstream (29/106, 27.36%), respiratory tract (25/106, 23.58%) (Fig. 1C). Notably, gram-negative bacteria were the predominant pathogens in cases of bloodstream infection, digestive tract infection, or skin/mucosa infection in patients with HM (Fig. 1D). Detailed clinical characteristics are summarized in Table S1.

**Fig. 1:**
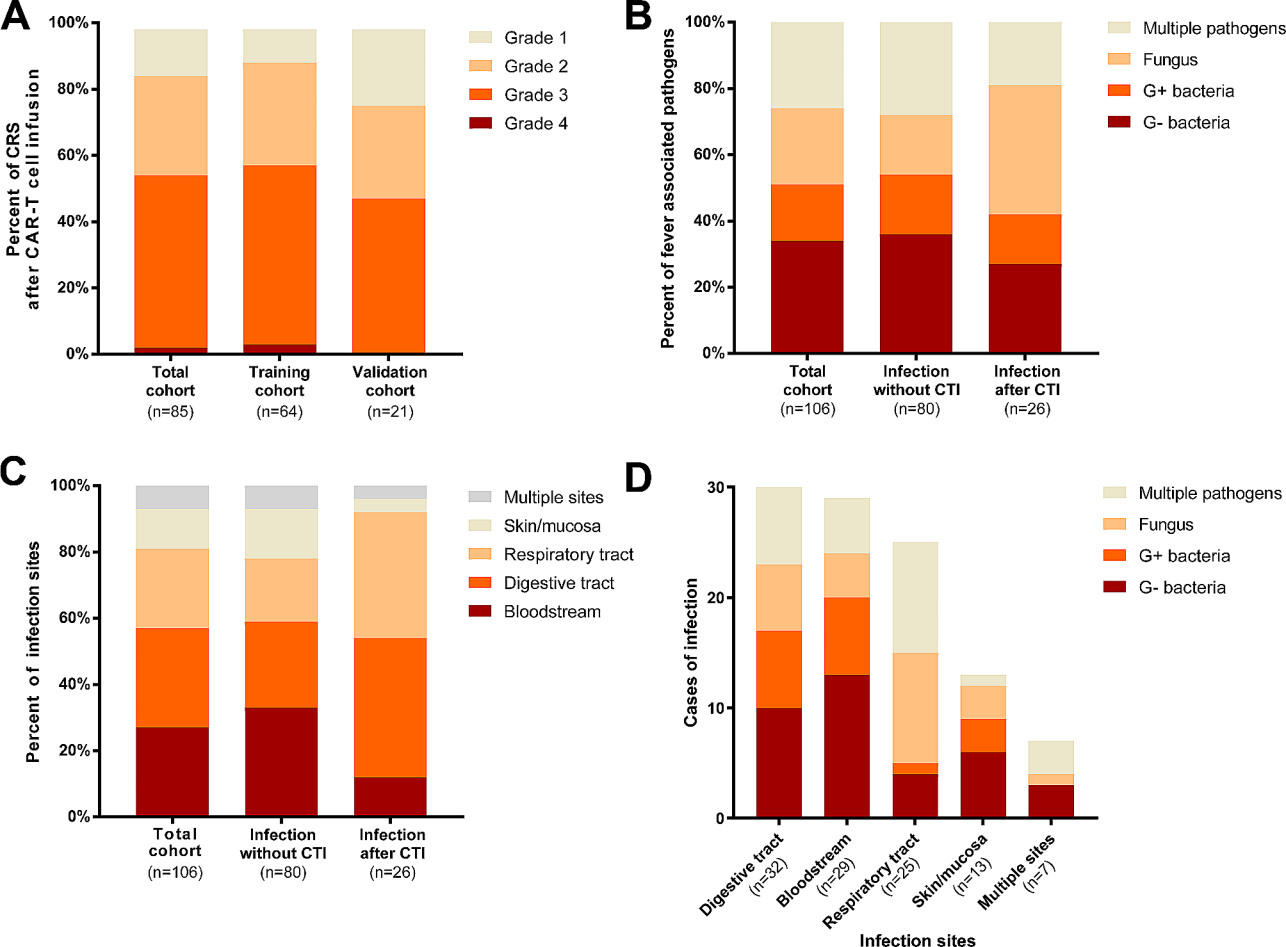
Overview of CRS and infection among patients with hematological malignancies. **A** Percent of febrile patients with different CRS grades in the total CRS cohort (*n* = 85), training cohort (*n* = 64) and validation cohort (*n* = 21). **B** Percent of infection cases induced by various pathogens in the total infection cohort (*n* = 106), and patient cohort with (*n* = 26) or without (*n* = 80) a history of CTI. **C** Percent of infections divided by different sites. **D** Number of infectious cases divided by different sites and pathogens

To construct the clinical model, patients without a potential overlap between infection and CRS (*n* = 165) were chronologically divided into training (*n* = 124) and validation (*n* = 41) cohorts at a ratio of approximately 3:1. Additionally, a separate cohort of patients with infectious fever after CTI (*n* = 26) was included in the validation cohort to assess the effectiveness of our models in distinguishing infectious fever in the context of CTI (Table S1).

A feasible decision tree model was obtained based on three cytokines with considerable accuracy. This model identified that IFN-β, CXCL1, and CXCL10 are key cytokines for the classification of febrile patients. Patients with ln(IFN-β) < 1.90 pg/mL, ln(CXCL10) > 5.66 pg/mL, and ln(CXCL1) < 8.20 pg/mL, were classified as having CRS or infection (Fig. 2A). The area under the curve reached approximately 0.942, and both sensitivity and specificity were no less than 90% in both the training and validation cohorts (Fig. 2B). Further analyses revealed that this model demonstrates a relatively higher potency in differentiating non-severe CRS, and equal efficiency in differentiating different types of infections (Fig. S1).

**Fig. 2:**
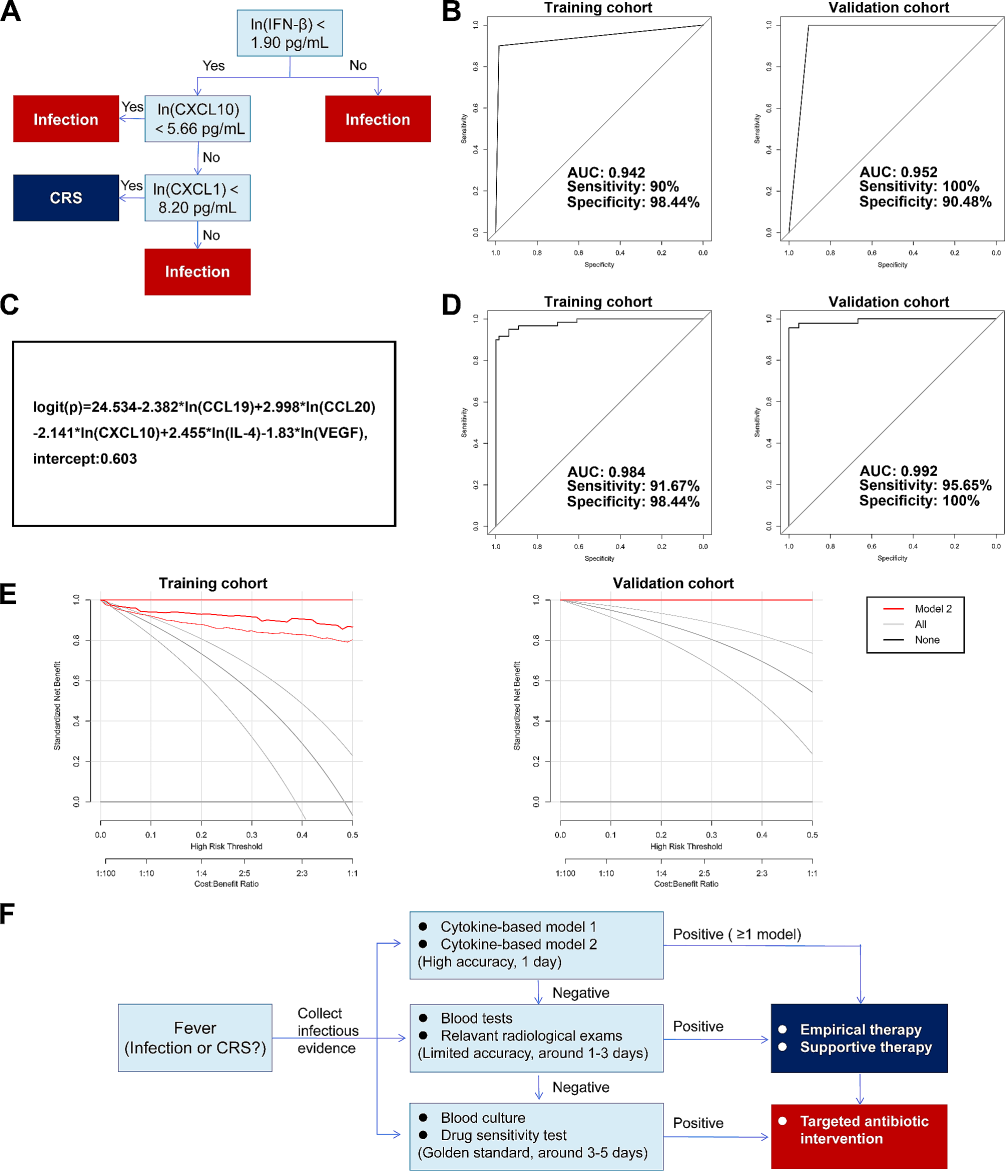
Construction and validation of cytokine-based clinical models for differentiating CRS-related fever from infectious fever. **A** Decision tree classification for differentiating between CRS and infection. **B** Receiver operating characteristic (ROC) curve of the decision tree classification in the training cohort and validation cohort. **C** An equation of clinical model for discriminating between CRS and infection. **D** ROC curve of stepwise logistic regression. **E** Decision curve analyses demonstrating the net benefit in training (left) and validation (right) cohorts. The black curve (“all”) represents that this model identifies that all the patients suffered from infectious fever, and the grey curve (“none”) represents that this model identifies that all the patients suffered from CRS-related fever. The upper and lower lines of each group represent a 95% confidence interval. **F** Flow chart of the optimized clinical management of fever after CAR-T cell infusion. AUC, area under the curve; CXCL, CXC motif chemokine ligand; IFN, interferon; IL, interleukin; VEGF, vascular endothelial growth factor

Further, a five-cytokine-based differentiation model built by a stepwise regression analysis demonstrates even higher accuracy (Fig. 2C-D). The logistic regression score was converted into a modeled probability. Febrile patients with a high probability (> 0.603) would be recognized as an “infection” case, while those with a lower score would likely have CRS. This model confirmed high levels of CXCL10, CCL19, and VEGF as indicators for CRS. Additionally, high levels of CCL20 and IL-4 were identified as indicators of infection. In the validation cohort, this model effectively distinguished between infectious and CRS cases with a high accuracy (Fig. 2D). Further analyses revealed that this model showed a slightly inferior ability to differentiate between CRS and fungal infections or digestive tract infections (Fig. S1D-F). Moreover, the results of decision curve analyses revealed that a benefit could be attained in a wide range of threshold probabilities by applying these models in clinical practice (Fig. 2E).

Given the limited timeliness of golden standards for fever differentiation, we built cytokine-based differentiation models considering the rapidity of cytokine detection assays. Besides practicability, the differential patterns could enlighten the further investigations into the underlying mechanisms (Table S2). Previously, Luo et al. proposed a combination of “double peaks of IL-6” pattern and a three-cytokine based prediction model for the rapid diagnosis of severe infection after CTI [11]. Additionally, Diorio et al. developed a classification model for differentiating sepsis and CRS in critically ill patients with high accuracy, utilizing IFN-γ and IL-1β [12]. Comparatively, our study, based on a large sample size with heterogeneous patients in aspect of disease types and CAR-T cell products, might harbor higher practicability and accuracy in clinical practice. Further, we integrated the novel differentiation models to the optimized clinical management flow for CAR-T cell therapy, which could promote the early diagnosis and intervention of the cause of fever (Fig. 2F).

### Electronic supplementary material

Below is the link to the electronic supplementary material.

Supplementary Material 1

## Data Availability

The data that support the findings of this study are available from the corresponding author upon reasonable request.

## Abbreviations

CAR: Chimeric antigen receptor
HMs: Hematological malignancies
CRS: Cytokine release syndrome
CTI: CAR-T cell infusion
r/r: relapsed or refractory
