# Supplementary material for: Cytokine-based models for efficient differentiation between infection and cytokine release syndrome in patients with hematological malignancies
Source: Exp Hematol Oncol. 2024 Mar 5;13:28. doi: 10.1186/s40164-024-00495-6 (PMC10913574; doi:10.1186/s40164-024-00495-6)
Supplement: Supplementary file 1 — Supplementary Material 1 [file 40164_2024_495_MOESM1_ESM.docx]

**Cytokine-based models for efficient differentiation between infection and cytokine release syndrome in patients with hematological malignancies**

Authors: Linqin Wang^*^, Yuqi Lv^*^, Linghui Zhou^*^, Shenghao Wu, Yuanyuan Zhu, Shan Fu, Shuyi Ding, Ruimin Hong, Mingming Zhang, Hanjing Yu, Alex H. Chang, Guoqing Wei, Yongxian Hu^#^, He Huang^#^

*These authors contributed equally to this work.

#Corresponding authors.

**Additional file: Detailed methods and results**

**METHODS**

***Patients.*** This study included 111 febrile patients with relapsed or refractory hematological malignancies who received chimeric antigen receptor (CAR)-T cell therapy (ChiCTR1800017404, ChiCTR-ORN-16008948, ChiCTR1800017402, ChiCTR1800015575, and NCT04599556) at our center. Among them, 85 developed cytokine release syndrome (CRS)-related fever, and 26 developed infectious fever after CAR-T cell infusion (CTI). Furthermore, 80 patients who developed infectious fever during conventional therapy at our center were included in this study. After obtaining approval from the Ethics Committee of the First Affiliated Hospital of Zhejiang University, this study was conducted in full compliance with the ethical principles of the Declaration of Helsinki.

All enrolled patients met the inclusion criteria as reported previously [1-4]. After obtaining fully informed consent, blood samples were collected from patients during the episode of fever (>38℃). For patients with CRS, blood was collected at the peak of CRS (around the peak of temperature). Meanwhile, for patients with infectious fever, blood samples were collected during both the peak and recovery stages. Serum samples were stored in the liquid nitrogen. Demographic and clinical characteristics of patients were obtained from medical records.

***CAR-T cell production and clinical protocol.*** After peripheral blood mononuclear cell apheresis, autologous T cells were activated and expanded using CD3/CD28 beads (40203D, Thermo Fisher Scientific, USA), followed by lentiviral transfection. Plasmids containing the second-generation CAR sequence with a 4-1BB costimulatory domain were used to envelope the lentiviral vector.

Prior to CTI, the patients received lymphodepletion therapy consisting of a three-day course of fludarabine and cyclophosphamide. During hospitalization, vital signs and blood tests were monitored on a daily basis after CTI.

***Grading of fever and CRS.*** Fever was graded according to the National Cancer Institute Common Terminology Criteria for Adverse Events version 5.0 (Table S3). Cases that were classified as grade 3-5 were considered “severe”.

Evaluation of the severity of CRS was carried out by three experienced clinicians, with any inconsistencies being addressed through further discussions. The criteria and grading of CRS were based on a revised grading system [5], with grade 3-4 CRS classified as “severe”.

***Definition of infection.*** The diagnosis of infection was established based on microbiological evidence, which was supported by relevant clinical symptoms and radiologic features. Microbiological evidence included positive blood cultures, sputum cultures, urine cultures, pharyngeal swabs, secretion examinations, and fecal examinations. Cases of multiple organism infections during the same episode of fever were defined as multiple infections.

***Cytokine detection assay.*** Batches of serum samples were restored to room temperature from being frozen in liquid nitrogen for cytokine detection. A panel of 44 cytokines was detected using a human 44-plex magnetic bead kit (LKTM014, R&D Systems, USA) on the Luminex platform. And forty-four cytokines were analyzed including CXCL1, CXCL2, CXCL10, CCL2, CCL3, CCL4, CCL11, CCL19, CCL20, CD40 ligand, EGF, FLT-3 ligand, FGF basic, G-CSF, GM-CSF, granzyme B, IFN-α, IFN-β, IFN-γ, IL-1α, IL-1β, IL-1ra, IL-2, IL-3, IL-4, IL-5, IL-6, IL-7, IL-8, IL-10, IL-12, IL-13, IL-15, IL-17A, IL-17E, IL-33, PDGF-AA, PDGF-AB/BB, PD-L1, RANTES, TRAIL, TGF-α, TNF-α, and VEGF. The serum cytokine detection process is shown in Fig. S2. Briefly, a microparticle cocktail was added to each well of the microplate, and then the samples were incubated with samples for 2 hours at room temperature on a horizontal orbital microplate shaker. After incubation, the samples were treated with a biotin-antibody cocktail for 1 hour at room temperature, followed by 30-minute incubation with streptavidin-PE reporter dye. The microparticles were ultimately resuspended, and tested using the *Luminex 200* analyzer (R&D Systems, USA).

***Statistical analyses.*** Continuous variables are described as medians with ranges, whereas categorical variables are described as percentages.

***Model development.*** During the construction of the decision tree models, 44 cytokines were used to discriminate febrile subjects from the training cohort as a member of the infection or CRS cohorts. The levels of cytokine were natural log-transformed before analysis. Key variables were selected to form nodes in classification tree using the “tree” package.

The equation model was derived from stepwise logistic regression using the “rms” package. Both baseline characteristics and cytokine levels were included in the univariable logistic analysis, followed by the selection of the least absolute shrinkage and selection operator (LASSO) regression model. Selected factors with non-zero coefficients in the LASSO regression were recognized as potential factors. These factors were then included in a stepwise multivariable logistic regression analysis. The models were fitted using minimum Akaike information criterion (AIC) values. Finally, the equation model was developed to determine the probability of an infectious fever. The logit(p) (score) of models was transformed into probability by the equation ($\text{p}\text{=}\frac{e^{score}}{e^{score}+1}$). Receiver operating characteristic (ROC) curves and decision curve analyses (DCA) were used to evaluate the accuracy and usefulness of the models.

The model construction was implemented using R software (version 4.2.2), and the data analysis was conducted using either IBM SPSS Statistics version 20 or R software. Figures were created using BioRender.com or Sangerbox Tools (*http://www.sangerbox.com/tool*).

**SUPPLEMENTARY TABLES**

**Table S1 Demographic and clinical characteristics of patients in training and validation cohorts**

| **Characteristic** | **Training cohort (n=124)** | | **Validation cohort (n=67)** | | |
| --- | --- | --- | --- | --- | --- |
|  | **CRS**  **(n=64)** | **Infection without CTI (n=60)** | **CRS**  **(n=21)** | **Infection without CTI (n=20)** | **Infection after CTI (n=26)** |
| **Age, years** | 55 [21-74] | 54 [16-83] | 61 [16-74] | 57 [22-82] | 58 [20-83] |
| **Male, n (%)** | 38 (59.38%) | 35 (58.33%) | 9 (42.86%) | 15 (75%) | 18 (69.23%) |
| **Disease, n (%)** |  |  |  |  |  |
| Multiple myeloma | 51 (79.69%) | 16 (26.77%) | 21 (100%) | 4 (20%) | 7 (26.92%) |
| Leukemia | 13 (20.31%) | 38 (63.33%) | / | 12 (60%) | 10 (38.46%) |
| Lymphoma | / | 6 (10%) | / | 4 (20%) | 9 (34.62%) |
| **Targets of CAR-T cell, n(%)** |  |  |  |  |  |
| BCMA | 51 (79.69%) | / | 21 (100%) | / | 7 (26.92%) |
| CD19 or CD22 | 10 (15.63%) | / | / | / | 15 (57.69%) |
| CD19/CD22 dual target | 3 (4.69%) | / | / | / | / |
| CD7 | / | / | / | / | 4 (15.38%) |
| **Peak temperature, ℃** | 39.80 [38.40-41.00] | 39.50 [38.20-41.00] | 39.5 [38.60-40.30] | 38.6 [38-39.9] | 39.7 [38.10-40.70] |
| **Peak fever grade, n (%)** |  |  |  |  |  |
| Grade 1 | 9 (14.06%) | 20 (33.33%) | 6 (28.57%) | 14 (70%) | 7 (26.92%) |
| Grade 2 | 31 (48.44%) | 29 (48.33%) | 12 (57.14%) | 6 (30%) | 11 (42.31%) |
| Grade 3-4 | 24 (37.50%) | 11 (18.33%) | 3 (14.29%) | 0 (0) | 8 (30.77%) |
| **Onset time of fever, days** | 1 [0-13] | / | 6 [1-17] | / | 4.5 [0-133] |
| **Duration of fever, days** | 8 [1-37] | 6 [1-49] | 5 [2-15] | 2 [1-10] | 8.5 [3-23] |
| **Blood tests during fever** |  |  |  |  |  |
| WBC, ×10^9^/L | 1.20 [0.03-12.20] | 0.7 [0.01-75.6] | 1.23 [0.06-13.55] | 0.76 [0.03-9.44] | 0.88 [0.05-6.72] |
| ANC, ×10^9^/L | 0.80 [0-10.90] | 0.48 [0-100] | 0.60 [0-11.64] | 0.17 [0-6.29] | 0.25 [0-4.85] |
| ALC, ×10^9^/L | 0.09 [0-4.70] | 0.3 [0-10.59] | 0.11 [0.01-2.11] | 0.39 [0.01-2.34] | 0.16 [0-1.5] |
| LDH, U/L | 220 [116-9340] | 228 [67-5029] | 263 [114-1693] | 196 [95-895] | 276 [135-3965] |
| CRP, ng/mL | 60.07 [2.40-285.20] | 38.70 [3.30-339.80] | 40.96 [0.10-134.80] | 39.78 [4.60-150.48] | 30.13 [0.26-361.21] |

Data were described as n (%) or median [range].

ANC, absolute neutrophil count; ALC, absolute lymphocyte count; CRP, C-reactive protein; CTI, CAR-T cell infusion; LDH, lactate dehydrogenase; WBC, white blood cell count.

**Table S2 Summary of the biological properties of cytokines involved in differentiation models**

| **Cytokine** | **Attribute** | **Function and possible mechanisms** | **Reference** |
| --- | --- | --- | --- |
| **CXCL1** | Positively associated with infectious fever | Recruit diverse immune cells to participate in the defense against infection | [6] |
| **CCL20** |  |  | [7, 8] |
| **IFN-β** |  | Amplify the immune responses | [9] |
| **IL-4** |  | Regulate excessive inflammation, and improve patients’ outcomes following sepsis | [10] |
| **CXCL10** | Positively associated with CRS-related fever | Elevate during the initiation and peak phases of CRS, involving in recruiting immune cells | [11] |
| **CCL19** |  |  | [12] |
| **VEGF** |  | A potent inducer of vascular permeability, which may be involved in capillary leak | [13, 14] |

**Table S3 Grading criteria for fever**

| **Symptom**  **Grade**  **Grade** | **Grade 1** | **Grade 2** | **Grade 3** | **Grade 4** | **Grade 5** |
| --- | --- | --- | --- | --- | --- |
| **Fever** | 38-39℃ | >39-40℃ | >40℃ within 24 hours | >40℃ longer than 24 hours | Deceased |

Criteria were defined per Common Terminology Criteria for Adverse Events (CTCAE) version 5.0.

**SUPPLEMENTARY FIGURES**

**
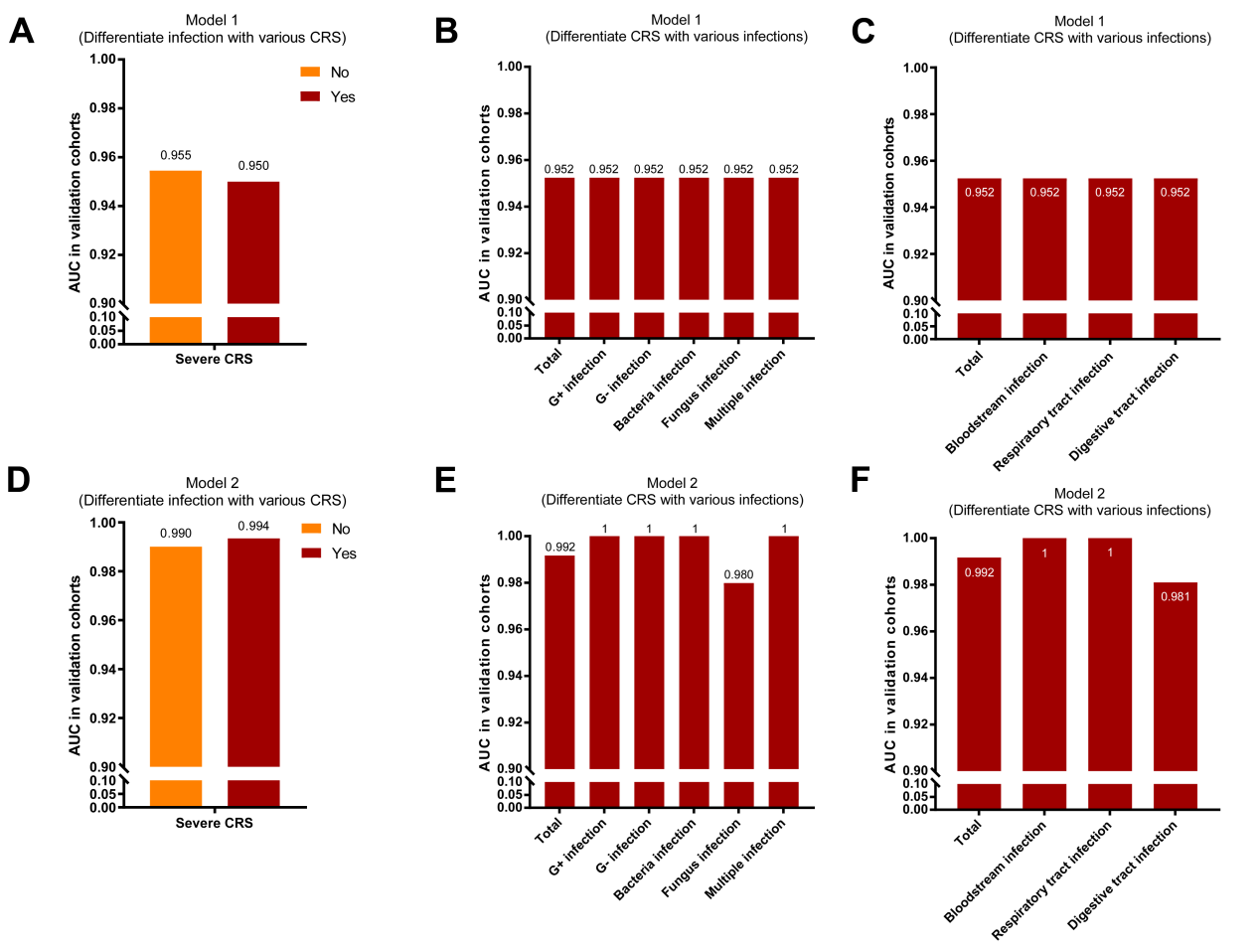
**

**Fig S1 The effect size of models in differentiating different types of CRS and infection. (A-C)** The AUCs of decision tree model (model 1) in differentiating different types of CRS, different-pathogen induced infection, and different-site infection. **(D-F)** The AUCs of equation model (model 2) in differentiating different types of CRS, different-pathogen induced infection, and different-site infection.

AUC, area under the curve; CRS, cytokine release syndrome.

**
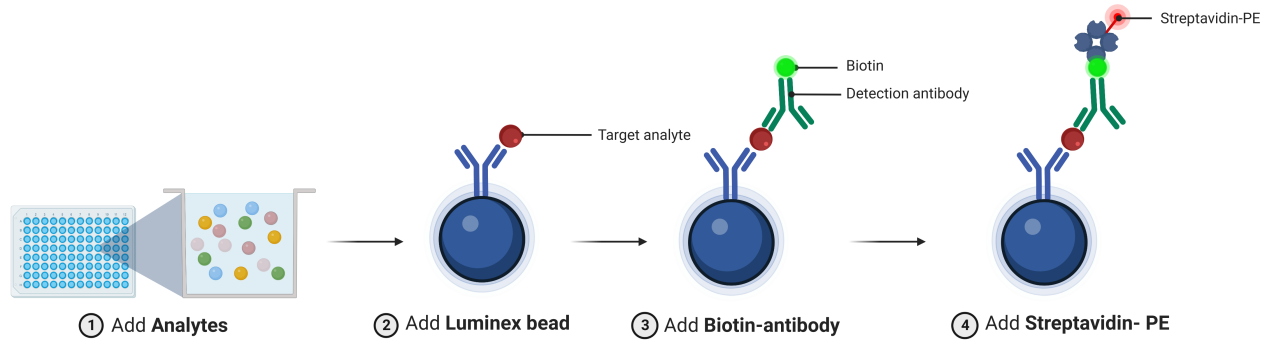
**

**Fig S2 Process of cytokine detection via luminex platform.** Microparticle cocktail was incubated with samples, followed by adding Biotin-Antibody cocktail and Streptavidin-PE reporter dye. The processed samples were analyzed by *Luminex 200* analyzer.

**SUPPLEMENTARY REFERENCES**

1. Song F, Hu Y, Zhang Y, Zhang M, Yang T, Wu W, et al. Safety and efficacy of autologous and allogeneic humanized CD19-targeted CAR-T cell therapy for patients with relapsed/refractory B-ALL. J Immunother Cancer. 2023;11(2).

2. Zhang M, Zhou L, Zhao H, Zhang Y, Wei G, Hong R, et al. Risk Factors Associated with Durable Progression-Free Survival in Patients with Relapsed or Refractory Multiple Myeloma Treated with Anti-BCMA CAR T-cell Therapy. Clin Cancer Res. 2021;27(23):6384-92.

3. Wang Y, Yang Y, Hong R, Zhao H, Wei G, Wu W, et al. A retrospective comparison of CD19 single and CD19/CD22 bispecific targeted chimeric antigen receptor T cell therapy in patients with relapsed/refractory acute lymphoblastic leukemia. Blood Cancer J. 2020;10(10):105.

4. Wei G, Zhang Y, Zhao H, Wang Y, Liu Y, Liang B, et al. CD19/CD22 Dual-Targeted CAR T-cell Therapy for Relapsed/Refractory Aggressive B-cell Lymphoma: A Safety and Efficacy Study. Cancer Immunol Res. 2021;9(9):1061-70.

5. Lee DW, Gardner R, Porter DL, Louis CU, Ahmed N, Jensen M, et al. Current concepts in the diagnosis and management of cytokine release syndrome. Blood. 2014;124(2):188-95.

6. De Filippo K, Dudeck A, Hasenberg M, Nye E, van Rooijen N, Hartmann K, et al. Mast cell and macrophage chemokines CXCL1/CXCL2 control the early stage of neutrophil recruitment during tissue inflammation. Blood. 2013;121(24):4930-7.

7. Klaus DA, Seemann R, Roth-Walter F, Einwallner E, Motal MC, Tudor B, et al. Plasma levels of chemokine ligand 20 and chemokine receptor 6 in patients with sepsis: A case control study. Eur J Anaesthesiol. 2016;33(5):348-55.

8. Chen S, Kuang M, Qu Y, Huang S, Gong B, Lin S, et al. Expression of Serum Cytokines Profile in Neonatal Sepsis. Infect Drug Resist. 2022;15:3437-45.

9. Rackov G, Shokri R, De Mon MA, Martinez AC, Balomenos D. The Role of IFN-beta during the Course of Sepsis Progression and Its Therapeutic Potential. Front Immunol. 2017;8:493.

10. Song GY, Chung CS, Chaudry IH, Ayala A. IL-4-induced activation of the Stat6 pathway contributes to the suppression of cell-mediated immunity and death in sepsis. Surgery. 2000;128(2):133-8.

11. Reschke R, Gajewski TF. CXCL9 and CXCL10 bring the heat to tumors. Sci Immunol. 2022;7(73):eabq6509.

12. Forster R, Davalos-Misslitz AC, Rot A. CCR7 and its ligands: balancing immunity and tolerance. Nat Rev Immunol. 2008;8(5):362-71.

13. Freyer CW, Porter DL. Cytokine release syndrome and neurotoxicity following CAR T-cell therapy for hematologic malignancies. J Allergy Clin Immunol. 2020;146(5):940-8.

14. Wang L, Astone M, Alam SK, Zhu Z, Pei W, Frank DA, et al. Suppressing STAT3 activity protects the endothelial barrier from VEGF-mediated vascular permeability. Dis Model Mech. 2021;14(11).
